# The Effect of a Multimodal Occupational Therapy Program with Cognition-Oriented Approach on Cognitive Function and Activities of Daily Living in Patients with Alzheimer’s Disease: A Systematic Review and Meta-Analysis of Randomized Controlled Trials

**DOI:** 10.3390/biomedicines9121951

**Published:** 2021-12-20

**Authors:** Min-Joo Ham, Sujin Kim, Ye-Ji Jo, Chisoo Park, Yunkwon Nam, Doo-Han Yoo, Minho Moon

**Affiliations:** 1 Department of Occupational Therapy, Konyang University, 158, Gwanjeodong-ro, Seo-gu, Daejeon 35365, Korea; ham841106@naver.com (M.-J.H.); yeji0528@naver.com (Y.-J.J.); 1130cs@naver.com (C.P.); 2 Department of Biochemistry, College of Medicine, Konyang University, 158, Gwanjeodong-ro, Seo-gu, Daejeon 35365, Korea; aktnfl3371@naver.com (S.K.); yunkwonnam@gmail.com (Y.N.); 3 Research Institute for Dementia Science, Konyang University, Daejeon 35365, Korea

**Keywords:** Alzheimer’s disease, meta-analysis, non-pharmacological intervention, occupational therapy, multimodal program

## Abstract

Non-pharmacological intervention, which includes a broad range of approaches, may be an alternative treatment for Alzheimer’s disease (AD). Multimodal non-pharmacological intervention alleviates cognitive dysfunction and the impairment of activities of daily living (ADL) in AD patients. However, it is still unclear which combination of non-pharmacological interventions is preferred. We selected a non-pharmacological intervention combined with occupational therapy (OT). We investigated the effect of a multimodal OT program with cognition-oriented approach on cognitive dysfunction and impairments of ADL in patients with AD. Four electronic databases were searched from January 2000 to August 2020. The studies were assessed for heterogeneity, quality assessment, effect size and publication bias. A total of seven randomized controlled trials examining multimodal OT programs with cognition-oriented approach in AD patients were included in the meta-analysis. Compared with the control group, the multimodal OT program with cognition-oriented approach group was statistically beneficial for cognitive dysfunction (95% CI: 0.25–0.91). However, compared with the control group, the multimodal OT program with cognition-oriented approach group tended to be beneficial for basic ADL, and instrumental ADL. These results suggest that the multimodal OT program with cognition-oriented approach might be the optimal multimodal non-pharmacological intervention for improving cognitive dysfunction in AD patients.

## 1. Introduction

Dementia is defined as a state in which daily living is severely disrupted by a combination of symptoms, such as memory impairment, aphasia, apraxia, agnosia, or impaired executive function caused by an acquired brain disease [1]. Alzheimer’s disease (AD) accounts for 55–70% of all dementia cases and is clinically characterized by gradually worsening severe memory loss that causes impairment of daily living [2]. Treatment approaches for patients with AD are divided into pharmacological and non-pharmacological approaches. Interestingly, according to the DSM-5 [3], ADL is mentioned as an important diagnostic criterion along with cognitive factors in the definition of dementia. Research has been expanding on non-pharmacological treatment as a strategy to improve the performance of activities of daily living (ADL) and maintain cognitive function in patients with AD [4,5]. Indeed, the number of studies on non-pharmacological interventions has rapidly increased in the last 15 years [6].

Non-pharmacological interventions include occupational therapy (OT), reminiscence therapy, reality orientation therapy, validation therapy, sensory stimulation, multisensory stimulation, art therapy, music therapy, aromatherapy, light therapy, physical activity, exercise, cognitive stimulation, cognitive training, and cognitive rehabilitation. In addition, there are four approaches to non-pharmacological interventions: (1) cognition-oriented, (2) emotion-oriented, (3) behavior-oriented, and (4) stimulation-oriented approaches. The cognition-oriented approach aims to improve cognitive dysfunction, the most prevalent symptom of AD. In particular, cognition-oriented approaches are widely used in combination with other non-pharmacological interventions, such as OT, to optimize cognitive function for ADL improvement [7,8,9,10]. Moreover, risk factors for AD include aging, cardiovascular disease, and diabetes, as well as socio-demographical variables, psychological variables, hearing loss, and anxiety [11]. Therefore, for AD treatment, not only treating the disease, but also non-drug interventions that can affect many aspects with a variety of approaches can be helpful.

Several studies have suggested the potential of OT in the treatment of AD [12,13,14,15]. OT seeks not only simply to treat the disease, but also to understand and respect patients with AD as human beings by applying a ‘social-psychological model’ [15,16]. These aspects are clearly different from pharmacological approaches or other non-pharmacological interventions. In particular, numerous studies show the beneficial effect of OT intervention on dementia patients [17,18,19]. Moreover, the meta-analysis about OT interventions based on sensory stimulation on dementia patients reveals the improvement of behavioral disorders of dementia patients [18]. Moreover, OT intervention to maintain body function was showed to improve the ADL ability of dementia patients [20]. One of the studies reported that OT-including cognitive interventions improved the abilities of AD patients to perform ADL [21]. Interestingly, multimodal OT interventions are used to alleviate occupational imbalances, which is the loss of balance while engaging in daily activities caused by dementia [4]. Moreover, in previous studies, multimodal OT programs with cognition-oriented approaches have been reported to have positive effects on cognitive function and ADL in patients with dementia [8,22]. Therefore, OT is strongly encouraged in the treatment of patients with mild-to-moderate dementia [23,24].

Previous studies have demonstrated the suitability and effectiveness of the multimodal OT program with cognition-oriented approach in patients with dementia [8,22]. However, to the best of our knowledge, no study has been conducted meta-analysis regarding the effectiveness of multimodal OT program with cognition-oriented approach. In this study, we performed a systematic literature review and meta-analysis to investigate whether the multimodal OT program with a cognition-oriented approach has any effects on cognitive dysfunction and ADL impairment, which are considered major problems for patients with AD. Our study could provide clinicians in the field of dementia with evidence of a multimodal OT program with a cognition-oriented approach.

## 2. Materials and Methods

### 2.1. Search Strategy to Identify Relevant Studies and Criteria for Inclusion

We conducted a search for studies using a multimodal OT program with cognition-oriented approach in patients with AD. Studies with publication dates between January 2000 and August 2020 were retrieved from the MEDLINE, CINAHL, PubMed, and Academic Search Complete databases. The main search terms were as follows: Alzheimer, Dementia, Intervention, Cognitive stimulation, Cognitive training, Cognitive rehabilitation, Multimodal, Multidisciplinary, Multistimulation, Occupational therapy, Cognitive function, Activities of daily living, and Randomized controlled trial.

The inclusion criteria were as follows: (1) studies on patients with AD; (2) studies on multimodal OT program with cognition-oriented approach; (3) studies measuring changes in overall cognitive function or ADL; (4) randomized controlled trials (RCTs); (5) studies published in English; and (6) studies that indicated the sizes of the intervention and control groups, the means and standard deviations, or the standardized mean difference (SMD) scores. The exclusion criteria were as follows: (1) graduation theses, (2) case studies, (3) literature reviews, (4) pilot studies that did not lead to the main studies, and (5) studies result on caregivers were excluded. The criteria for multimodal OT program with cognition-oriented approach studies as follows: (1) studies that applied cognitive stimulation, cognitive training, and cognitive rehabilitation interventions simultaneously, clearly including OT, and (2) studies involving occupational therapists.

### 2.2. Data Extraction

**Figure 1:**
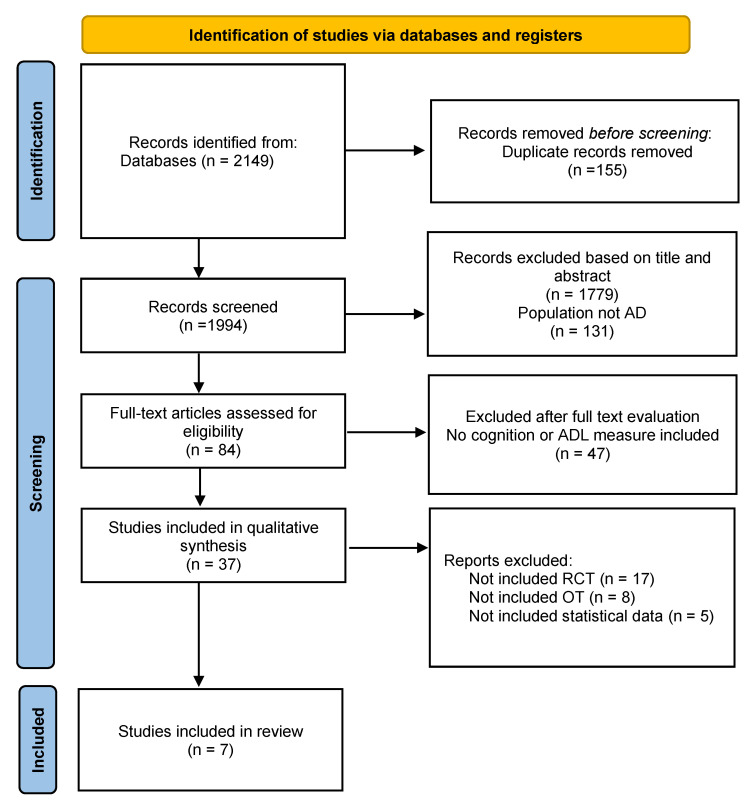
Flow diagram of the study selection process.

Based on the Preferred Reporting Items for Systematic reviews and Meta-Analysis (PRISMA) [25], we have diagrammed the step-by-step process of study selection in a flowchart (Figure 1) (This review was not registered in PRISMA). A total of 2149 studies were retrieved from the initial literature search, and 155 duplicate studies were excluded. Of the remaining 1994 studies, 1779 studies were excluded based on the inclusion/exclusion criteria by reviewing the titles and abstracts, and 131 studies were excluded because they did not include patients with AD. The full texts of the remaining 84 studies were inspected, and 47 studies were excluded because they did not assess cognition or ADL. Finally, studies that were non-RCTs that did not thoroughly describe the characteristics of OT, or that did not show means or SMD were excluded, and the remaining seven studies were selected for the final analysis.

During data selection and review, two researchers performed the literature search in independent locations and completed the prepared results. If there was any disagreement between the researchers during this process, a consensus result was obtained by a research meeting in which the researchers reviewed the main text together.

### 2.3. Evidence Table Construction

In this study, we classified the characteristics of the seven selected RCTs based on the PICOTS (patient, intervention, comparison, outcome, time, and setting/study design) criterion. The results of patients with AD were completed in the following order: sex and age, sample sizes, intervention methods, number of treatment sessions, location of treatment sessions, and instruments used to assess outcomes (Table 1).

ADAS-Cog, Alzheimer’s Disease Assessment Scale-Cognitive subscale; ADLs, Activities of daily living; BADL, Basic activities of daily living; CG, Control group; EG, Experimental group; COPM, Canadian occupational performance measure; CR, Cognitive rehabilitation; DSM-4, Diagnostic and Statistical Manual of Mental Disorders; ICD-10: International Classification of Diseases; IADL, Instrumental activities of daily living; ILS, Independent living scale; K-MMSE, Korean version of mini mental status examination; MBI, Modified Barthel index; MCP, Multidomain cognitive program; MIP, Multicomponent intervention program; MMSE, Mini mental state examination; MRI, Multimodal rehabilitative intervention; MRP, Multidisciplinary rehabilitation program; NINCDS-ADRDA: National Institute of Neurological and Communicative Disorders and Stroke; N/A, Not assessment; N/I, Not intervention; OT, Occupational therapy; RDRS-2, Rapid Disability Rating Scale-2

### 2.4. Assessing the Quality of Research

Cochranés risk of bias (ROB) is an instrument used to evaluate potential bias that can occur in the design of RCTs. The ROB assesses seven domains: random sequence generation, allocation concealment, blinding of participants and researchers, blinding of outcome assessors, incomplete outcome data, selective outcome reporting, and other potential sources of bias that threaten the validity [32]. The results of quality assessment can be classified according to the Scottish Intercollegiate Guidelines Network levels of evidence [33]. In this study, the quality assessment was performed independently by two researchers, and when the results differed, the final quality grade was determined through discussion.

### 2.5. Statistical Analysis

For meta-analysis, we performed data coding and analysis using R version 4.1.2 meta and metafor package [34] and RevMan 5.3, provided by the Cochrane collaboration. We performed a meta-analysis using the change value of the mean score following interventions, the change value in the SMD following the interventions, and the group size for the test and control groups in each study. If the study provided the change value of the mean or SMD following the interventions, the raw data were used without any changes. If the change value was not provided, the change was calculated from the final value [35].

#### 2.5.1. Statistical Heterogeneity

For statistical heterogeneity, Cochran’s Q-test (chi-square test) and Higgins I^2^-statistic were used to verify the homogeneity of the groups with regard to effect size in the results of each study. When the Q-test’s *p*-value is ≥0.1, a fixed-effects model can be used, but when it is <0.1, a random-effects model can be used [35]. For the Higgins I^2^-statistic, I^2^ < 50% indicates that a fixed effects model can be used, while I^2^ > 50% indicates that a random-effects model can be used [36].

#### 2.5.2. Calculation of Effect Size

The effect size was calculated through the comparison of dependent variables between the test group and the control group of the seven RCTs studies selected, and the change in mean and SMD and the number of participants were used according to Cohen’s interpretation criteria [37]. In the interpretation of the overall effect size, ≤0.2 is considered a small effect, around 0.5 is considered a moderate effect, and ≥0.8, is considered a large effect [37]. The confidence interval (CI) was set at 95% of SMD.

#### 2.5.3. Publication Bias

We constructed a contour-enhanced funnel plot using the metafor package for meta-analyses in R [34] to statistically test for the risk of publication bias. A contour-enhanced funnel plot suppresses non-significant results and is known to be more effective at detecting publication bias [38]. In accordance with Sterne and Egger [39], the vertical axis was labeled as the standard error, and the horizontal axis as the observed outcome. Additionally, the statistical significance of publication bias was calculated using fail-safe N statistics.

## 3. Results

### 3.1. Included Studies Characteristics

#### 3.1.1. Diagnostic Criteria and Research Design

The seven studies selected involved participants with AD, and all studies had an RCTs design. All subjects (100%) were patients with AD. The six studies used diagnostic criteria of NINCDS-ADRDA 4 (57%), DSM-4 1 (14%), and ICD-10 1 (14%). One study did not support the detailed information on the diagnostic criteria.

#### 3.1.2. Demographic of Study Participants

A total of 453 participants were included in the meta-analysis. The countries where the study was conducted were Korea (28%), Italy (14%), Germany (14%), Brazil (14%), and Spain (14%). The mean age was ≥70 years and <80 years in six studies, and ≥60 years and <70 years in one study. Six studies presented the mean age separately for the test and control groups, and one study showed only the overall mean age.

#### 3.1.3. Intervention Type

Five studies applied an OT-based combination intervention program, but also included other treatment modalities, and two studies implemented a combination intervention program using only OT. The primary outcomes were cognitive function and ADL, with six studies including cognitive function, four studies including BADL, and three studies including IADL. Other therapeutic characteristics regarding the times or settings are described in detail in Table 1.

### 3.2. ROB Quality Assessment

**Figure 2:**
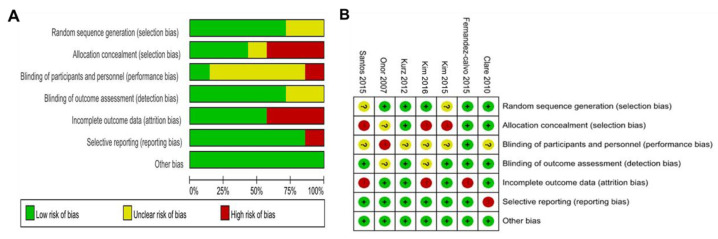
Risk of bias assessment across randomized controlled trials. Risk of bias graph (**A**) and summary (**B**).

In the Cochrane ROB analysis, there were three studies that showed a moderate ROB and four studies that showed a high ROB. Among the subdomains, random sequence generation was used in all studies, but two studies did not clearly describe their methods of random sequence generation. Second, three studies did not use allocation concealment, and in one study, the concealment method was unclear. Third, in terms of blinding of participants and researchers, there was one study that performed blinding, five studies did not discuss this matter, and one study did not perform blinding. Fourth, although most studies performed blinding of outcome assessors, two studies did not clearly report theirs. Fifth, in terms of incomplete outcome data, four studies were judged to have a low ROB due to missing or insufficient measurements, and three studies were judged to have a high ROB due to differences in the number of assessed participants in the test and control groups. Sixth, in terms of selective outcome, only one study was judged to have a high ROB due to insufficient information about the planned intervention process. Seventh, all the studies were judged to have a low risk of other sources of bias, threatening the validity of the study (Figure 2).

### 3.3. Meta-Analysis for the Effect of Multimodal OT Program with Cognition-Oriented Approach

#### 3.3.1. Meta-Analysis of the Effect of a Multimodal OT Program with the Cognition-Oriented Approach on Cognitive Decline in Patients with AD

**Figure 3:**
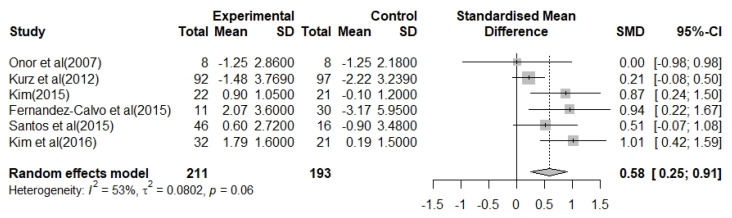
Forest plot for the effects of the multimodal occupational therapy program with cognition-oriented approach on cognitive decline in patients with AD.

Across six studies, 211 participants estimated the effect on cognitive decline in patients with AD. The heterogeneity for the overall effect size was I^2^ = 53% (*p* = 0.06), and a random-effects model was used. The overall effect of multimodal OT program with cognition-oriented approach on cognitive dysfunction was moderate and statistically significant, 0.58 (95% CI: 0.25–0.91). In addition, the effect size was positive (+) in five of the six studies. In summary, compared to controls, the multimodal OT program with the cognition-oriented approach showed significant improvement in cognitive dysfunction in patients with AD (Figure 3).

#### 3.3.2. Meta-Analysis of the Effect of a Multimodal OT Program with Cognition-Oriented Approach on BADL in Patients with AD

**Figure 4:**
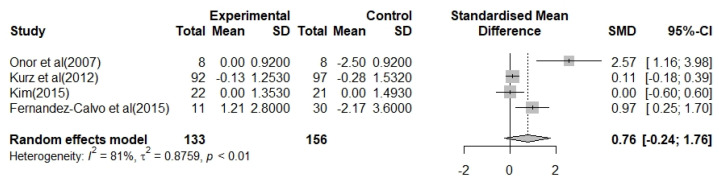
Forest plot for the effects of the multimodal occupational therapy program with cognition-oriented approach on BADL in patients with AD.

Four studies that reported 133 participants analyzed the effect on BADL in patients with AD. The heterogeneity for the overall effect size indicated I^2^ = 81% (*p* < 0.01), and a random-effects model was used. The effect size of multimodal OT program with cognition-oriented approach on BADL was moderate but not statistically significant, 0.76 (95% CI: −0.24–1.76). The effect size was positive in three of the four studies. However, one study did not show a positive effect. In total, compared to controls, the multimodal OT program with cognition-oriented approach group revealed a trend toward improving the impairment of BADL in patients with AD (Figure 4).

#### 3.3.3. Meta-Analysis of the Effect of a Multimodal OT Program with Cognition-Oriented Approach on IADL in Patients with AD

**Figure 5:**
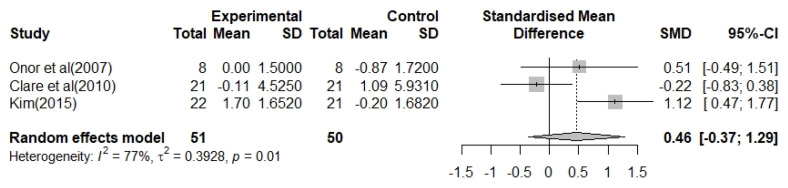
Forest plot for the effects of the multimodal occupational therapy program with cognition-oriented approach on IADL in patients with AD.

Three studies that investigated 51 participants were available for IADL outcomes. The heterogeneity of the overall effect size was I^2^ = 81% (*p* < 0.01), and a random-effects model was used. The effect size of multimodal OT program with cognition-oriented approach on IADL was moderate but not statistically significant, 0.46 (95% CI: −0.37–1.29). The effect size was positive in two of the three studies. However, one study showed a negative effect. Collectively, compared to controls, the multimodal OT program with cognition-oriented approach group showed trends for maintenance, improvement, or degradation of IADL in patients with AD (Figure 5).

### 3.4. Publication Bias

**Figure 6:**
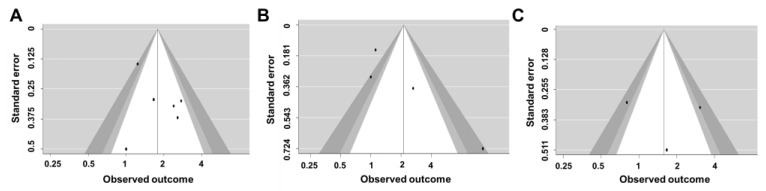
Funnel plot of the results of the studies included (**A**) funnel plot of cognitive function, (**B**) funnel plot of BADL, and (**C**) funnel plot of IADL.

We conducted a publication bias analysis to test the validity of all the study results (Figure 6). Since there were fewer than 10 studies included in our sample, we used a contour-enhanced funnel plot to visually inspect the left-right symmetry of the distribution of effect estimates. The aim of the method is to visually investigate the presence of small-study effects and to determine whether there is a relationship between precision (vertical axis) and effect size (horizontal axis) among the individual studies included in the meta-analysis [40]. In the assessment of cognitive function, six studies showed a symmetrical distribution. In addition, for BADL, three studies showed a relatively symmetrical distribution, but one study was located in the missing data region. For IADL, the three studies showed asymmetrical distribution. As a result of fail-safe N statistical analysis, publication bias was not observed as 94 in the case of cognitive function. Unfortunately, it was confirmed that publication bias exists with the safety coefficients (fail-safe N) of BADL and IADL of 30 and 25.

## 4. Discussion

In this study, we examined the effect of a multimodal OT program with cognition-oriented approach on cognitive dysfunction and impairment of ADL in patients with AD using meta-analysis. Seven RCT studies analyzed the effect size and heterogeneity of cognitive decline and impairment of BADL and IADL. In addition, to improve the reliability of the results, we assessed the quality of the selected studies and tested for publication bias. Cochrane’s ROB results suggested that selected studies have a relatively high level of quality, and the intervention effect can be very close to the actual intervention effect [41] (Figure 2). Furthermore, our study results showed that the multimodal OT program with cognition-oriented approach has beneficial effects on cognitive dysfunction and impairment of BADL and IADL in patients with AD (Figure 3, Figure 4 and Figure 5). These results may provide occupational therapists and dementia-related professionals with clinical evidence to support the use of non-pharmacological interventions.

Some previous studies have reported that combining two or more non-pharmacological interventions is more likely to slow cognitive decline more effectively than a non-pharmacological intervention alone [42]. Among the various combinations of multimodal non-pharmacological interventions, we investigated the effect of a multimodal OT program with cognition-oriented approach that seeks a patient-centered approach. The results of the meta-analysis demonstrated that the multimodal OT program with cognition-oriented approach had a significant effect on the improvement of cognitive dysfunction in patients with AD (Figure 3). Surprisingly, our results are consistent with a previous study that showed that multimodal intervention combined with OT, such as art therapy, language therapy, physical training, and computer-based cognitive training, for mild AD patients at day-hospital facilities which help to improve cognitive dysfunction [43]. Furthermore, although there was no statistical difference, the results of the meta-analysis indicated that the multimodal OT program with cognition-oriented approach tended to improve BADL disability in patients with AD (Figure 4). Interestingly, we found that the studies that provided the interventions one to three times for 16 weeks showed a greater improvement in BADL than studies that provided the interventions once per week for 8 weeks. In rehabilitation therapy for patients with neurodegenerative diseases, repetition training can be considered the most important factor for learning and recovery of skills [44]. Thus, we found that increasing the duration and frequency of the multimodal OT program with cognition-oriented approach helped to positively influence the BADL of patients with AD. Unfortunately, we did not find robust evidence that the multimodal OT program with cognition-oriented approach improved IADL disability (Figure 5), nor did we identify enough studies reporting on the outcomes of IADL to synthesize the results in a meta-analysis. We speculated that the various assessment tools used to measure IADL influenced the statistics. Moreover, these results have concerns that a small sample size, short treatment period, various treatment environments, and low sensitivity of evaluation tools may appear as methodological problems due to the nature of non-pharmacological intervention studies. To supplement these limitations in future meta-analyses, it will be necessary to focus on studies using the same assessment instruments, or to include additional related studies to increase the number of studies analyzed.

The reduction of various experiences and engagement, such as social networks, mental activity, and exercise, due to aging and disease, is associated with cognitive decline and the onset or progression of AD [45,46]. The increase in experience, engagement, and various stimuli through non-pharmacological intervention provides an opportunity for the brain to efficiently utilize and recruit existing neural networks and alternative neural networks [47,48,49]. One of the possible mechanisms by which non-pharmacological interventions, including OT, may affect AD could be related to cognitive reserve. In particular, cognitive reserve is the one of the important concepts of brain changes in neurodegenerative diseases, such as AD [50]. It is well known that in the AD brain reduces the number of neural precursor cells and decreases the generation of new neurons, causing a deficiency in hippocampal plasticity and cognitive reserve [51,52]. Interestingly, an increase in experience through non-pharmacological stimulus enhanced the rate of hippocampal progenitor cell proliferation in AD transgenic mice compared with that in the control group [53]. Furthermore, an increase in experience restored spatial memory in APP transgenic mice compared to the control group [54]. Thus, we suggest that experience-induced cognitive reserve through the complexity or novelty of non-pharmacological stimuli could be affected by AD-related cognitive dysfunction.

To the best of our knowledge, this is the first systematic review and meta-analysis of RCTs investigating multimodal OT program with cognition-oriented approach in patients with AD. Our findings will be able to provide practical information about non-pharmacological interventions for dementia patients. This study is useful because we investigated clinical evidence for the effects of OT, which is a representative non-pharmacological treatment modality for patients with dementia, even among other combination cognitive interventions. OT aims to help the patient participate in valuable tasks and perform significant ADL; to this end, it improves cognitive function and ADL, and can ultimately help to improve the patient’s quality of life [55]. For this reason, when non-pharmacological interventions are provided in a clinical setting to improve quality of life in patients with dementia, a multimodal OT program with cognition-oriented approach can be suggested as an effective therapeutic strategy for patients with AD. In particular, given that there was a statistically significant effect size for cognitive function and there was no publication bias on a scale that would result in an obvious error, we believe that the findings of this meta-analysis can provide highly objective and reliable clinical information. Nevertheless, our study had several limitations. First, I^2^, a value representing the heterogeneity of the overall effect size, was high (≥50%). Given that statistical heterogeneity was observed, we looked for the possible source of the variance and determined whether it was appropriate to combine studies [56]. Thus, we selected the random-effects model in this study. Moreover, to reduce heterogeneity, we also considered various factors, such as dementia type, intervention methods, and dependent variables, in the study selection stage. However, due to the overall low number of studies collected, it was not possible to control the same types of assessment instruments. In future studies, it will be necessary to expand the scope of data collection to include non-English and gray literature. Moreover, if a meta-analysis that was limited in scope to a specific assessment instrument was conducted, we believe that it would be possible to reduce the problems of heterogeneity that we observed. Second, although it is possible to combine effect sizes as long as there are two or more studies, for more accurate results, it is recommended to combine at least five studies [36]. In our analysis, while there were enough studies for cognitive function, for the outcomes of BADL and IADL, we had to conduct a meta-analysis using only three to four studies, which is not sufficient. In addition, publication errors were observed in the areas of BADL and IADL. However, in the results of this study, BADL and IADL did not show statistically significant effects. Thus, we speculate that the results of BADL and IADL will vary significantly not depending on the presence or absence of publication bias. Thus, the results need to be interpreted with caution, considering not only the possibility of publication bias but also a small number of studies. Therefore, it is necessary to accurately identify the publication bias even with a small amount of literature by utilizing various methods to check the publication bias [57]. If further studies are conducted on this topic, we propose to increase the number of experimental studies examining the effects of a multimodal OT program with cognition-oriented approach on ADL disability in patients with dementia. Third, since there were few studies available to compare the changes following the multimodal OT program with cognition-oriented approach, we were not able to show the extent of changes in the dependent variables with differences in the intervention schedule, time of assessment, or the intervention environment. Fourth, although the evaluation of the quality of research using ROB is high, among the seven ROB subdomains, allocation concealment and blinding of participants and researchers showed a high or uncertain risk of bias in more than half of the studies. For this reason, although the data in this meta-analysis show a high level of evidence because of the use of RCT designs, given the ROB, careful interpretation is required for actual clinical applications. Finally, we cannot exclude the possibility that studies on this research topic might have been missed during the literature search and selection process, and thus the results of this meta-analysis should be interpreted with caution.

## 5. Conclusions

In this study, to investigate the effects of a multimodal OT program with cognition-oriented approach for patients with AD, which is the most common form of dementia, we performed a systematic review analyzing the characteristics of the participants and quality of research. We performed a meta-analysis to examine the effects of a multimodal OT program with cognition-oriented approach on cognitive dysfunction and ADL disability in patients with AD. The results of the meta-analysis showed a statistically significant improvement in cognitive dysfunction. In addition, although the measured values of BADL and IADL did not show statistically significant results, the results of each study showed a tendency to maintain and improve cognitive function. Based on the above results, we propose that OT can be used very effectively as part of a combination intervention to improve cognitive dysfunction in patients with AD. In future research, it will be necessary to classify OT methods in detail depending on the stage of dementia progression, and to strictly control the factors, such as the assessment instruments and the intervention duration.

## Figures and Tables

**Table 1:** Characteristics of included studies.

	Study	Diagnosis Criteria	Participants				Intervention/Comparison			Time (Duration/Frequency)	Setting	Measurement of Outcomes	
			Country	Gender	Age (M ± SD) EG CG	EG (n) CG (n)	Intervention Format	Experimental Group	Control Group			Cognitive Function	ADLs
1	Onor et al., 2007 [5]	DSM-4	Italy	Male Female	68.0 ± 6.5 72.0 ± 5.2	8 8	Group	MRI (including OT)	N/I	1 h /1 session per weeks /16 weeks	Clinical psychiatry Visiting patient’s home	MMSE	BADL IADL
2	Clare et al., 2010 [26]	NINCDS-ADRDA	United Kingdom	Male Female	77.7 (Average)	22 22	Individual	OT based CR	N/I	1 h /1 session per weeks /8 weeks	Community based setting	N/A	ILS (IADL)
3	Kurz et al., 2012 [27]	ICD-10	Germany	Male Female	72.4 ± 8.5 75.0 ± 7.0	92 97	Individual	4 thematic modules: neurorehabilitation & psychotherapy (Including OT)	N/I	1 h /1 session per weeks /12 weeks	Hospital	MMSE	B-ADL
4	Kim, 2015 [28]	N/I	South Korea	Male Female	70.4 ± 7.9 71.4 ± 8.2	22 21	Group Individual	OT based CR	Watching videos Conversation with the examiner	1 h /1 session per weeks /8 weeks	No information	MMSE	MBI (BADL) COPM (IADL)
5	Kim et al., 2016 [29]	NINCDS-ADRDA	South Korea	Male Female	78.4 ± 1.0 78.5 ± 1.7	32 21	Group	MCP (including OT)	Routine pharmacotherapy	5 h /1 session per weeks /24 weeks	Regional dementia center	K-MMSE	N/A
6	Santos et al., 2015 [30]	NINCDS-ADRDA	Brazil	Male Female	75.7 ± 5.6 74.8 ± 4.7	46 16	Group	MRP (including OT)	N/I	5 h /2 sessions per weeks /12 weeks	Regional dementia center	MMSE	N/A
7	Fernandez-Calvo et al., 2015 [31]	NINCDS-ADRDA	Spain	Male Female	74.3 ± 3.9 72.3 ± 3.7	25 30	Individual	MIP (including OT)	N/I	90 min /3 sessions per week /16 weeks	Home	ADAS-Cog	RDRS-2 (BADL)
